# Time will tell: Decision making in premanifest and manifest Huntington’s disease

**DOI:** 10.1002/brb3.1843

**Published:** 2020-09-26

**Authors:** Beatrice Heim, Marina Peball, Carsten Saft, Sarah Maria von Hein, Philipp Ellmerer, Johanna Maria Piater, Klaus Seppi, Atbin Djamshidian

**Affiliations:** ^1^ Department of Neurology Medical University of Innsbruck Innsbruck Austria; ^2^ Department of Neurology Huntington – Center NRW St. Josef‐Hospital Ruhr‐University Bochum Bochum Germany

**Keywords:** decision making, Huntington's disease, information sampling, jumping to conclusions, perceptual decision making

## Abstract

**Objective:**

To investigate cognitive flexibility in premanifest and manifest Huntington's disease (HD).

**Background:**

HD is an autosomal dominant neurodegenerative disease characterized by motor, cognitive, and behavioral abnormalities with typical motor symptoms. In this study, we wanted to assess decision making in premanifest (pre‐HD) and manifest HD patients.

**Methods:**

A total of 77 non‐demented subjects including 29 pre‐HD, 22 manifest HD patients, and 26 healthy controls (HC) were included. We stratified the pre‐HD group based on their estimated years to disease onset into a far (FAR, *n* = 13) and a near (NEAR, *n* = 16) group. Furthermore, participants performed the Montreal cognitive assessment battery (MoCA), the trail making task part A and B (TMT A, TMT B), the Symbol digit modalities test (SDMT), and the beads task.

**Results:**

In the beads task, HD patients gathered less information than all other groups (all *p*‐values < .001). Furthermore, the NEAR group gathered less information than the FAR group (*p* < .001) and HC (*p* = .001). There was no difference between the HC and the FAR group (*p* = 1.0). In the TMT and the SDMT, HD patients were slower than all other groups (all *p*‐values < .01) but there were no other significant differences.

**Conclusions:**

Decision making with a higher degree of uncertainty may be an early neuropsychological sign to indicate the disease process prior to reaching criteria for motor diagnosis of HD.

## Graphical abstract

In this study, we assessed decision making in manifest Huntington's disease (HD) and premanifest HD patients. We could show that jumping to conclusions is an early marker of cognitive dysfunction in premanifest HD patients prior to reaching criteria for the motor diagnosis of HD.

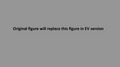

## 1 INTRODUCTION

Huntington's disease (HD) is an autosomal dominant neurodegenerative disease caused by an expansion of CAG trinucleotides on chromosome 4 and is characterized by motor, cognitive, and behavioral abnormalities and mood disturbances such as depression, apathy, and irritability (Conneally, 1984). Although clinical diagnosis of HD is based on motor symptoms, deterioration of executive functions is frequently observed prior to the onset of motor signs (Beste et al., 2012; Ho et al., 2003; Kirkwood, 2000; Watkins, 2000). Several studies have shown that HD is associated with deficits in decision making, which could explain the increased presence of dysfunctional behavior such as increased risk taking (Eddy & Rickards, 2012; Kalkhoven et al., 2014). Furthermore, impaired decision making, cognitive decline, obsessive behavior, and deficits in inhibitory control have been reported in HD mutation carriers without distinct motor symptoms (Beglinger et al., 2008; D'Aurizio et al., 2019; De Lucia, Peluso, Roca, De Michele, et al., 2019; Julio et al., 2019; Migliore et al., 2019; Paulsen & Long, 2014; Stout et al., 2011). Neuropsychological tasks assessing frontal executive functions such as the Symbol Digit Modalities Test, the Stroop Test, the Trail Making Test, and tasks requiring mental flexibility have been described to detected early impairment in HD mutation carriers (McGarry & Biglan, 2017; O'Rourke et al., 2011). A further study combined a Stroop Task and a Set‐shifting Task and detected changes in behavioral and neurophysiological measures sensitive for disease progression in HD mutation carriers 16 years prior to expected disease onset over a period of 6 months (Beste et al., 2013). The PREDICT‐HD study showed a worse performance in emotion recognition even in mutation carriers far from estimated disease onset (Stout et al., 2011).

In this study, we investigated cognitive performance in HD patients and HD mutation carriers far and near to estimated disease onset using a decision making task, as frontostriatal dysfunctions are known to affect emotional and cognitive processes which are critical for decision making. We used the beads task, which is an information sampling task to assess how much information patients gather before making a decision. Functional magnetic imaging studies have shown that the beads task activates brain areas that are typically affected in HD (Della Nave et al., 2010; Furl & Averbeck, 2011; Hamilton, 2003; Seo, Lee, & Averbeck, 2012).

Furthermore, several studies have shown that the beads task is sensitive to detect early impairment in decision making in patients with Parkinson's disease (Djamshidian et al., 2012; Djamshidian, O'Sullivan, et al., 2013), substance abuse (Djamshidian, Sanotsky, et al., 2013), and restless legs syndrome (Heim et al., 2017).

In addition, we conducted a Trail making test (TMT) and SDMT, since previous studies showed deficits in inhibitory control in HD and patients with prodromal HD (De Lucia et al., 2013; De Lucia, Peluso, Roca, Russo, et al., 2019; O'Rourke et al., 2011).

We hypothesized that manifest HD patients would perform worse than all other groups and that premanifest mutation carriers near to disease onset would perform worse than HC and mutation carriers far to disease onset.

## 2 Methods

The study was approved by local ethics committee of the Medical University of Innsbruck, Austria, and all participants provided written informed consent according to the declaration of Helsinki.

### 2.1 Study population

Twenty‐two manifest HD patients and twenty‐nine preclinical mutation carriers (pre‐HD) with a total motor score (TMS) of 5 or lower and a diagnostic confidence score (DCS) less than 4 on the Unified Huntington's Disease Rating Scale (UHDRS) were included were consecutively recruited (Unified Huntington's Disease Rating Scale, 1996). At time of assessment, no patient participated in a clinical trial other than registry studies. Results were compared to twenty‐six healthy controls (HC), who were also consecutively recruited from hospital staff or caregivers of patients with a variety of different neurological diseases other than HD at our outpatient department of the Department of Neurology, Medical University of Innsbruck, Austria.

Using the CAG repeat length and age‐based survival analysis of Langbehn and colleagues (Langbehn et al., 2004), we stratified the premanifest group into two groups (Stout et al., 2011): near to predicted disease onset (<15 years; NEAR) and far from predicted disease onset (>15 years; FAR). This value also corresponded to the baseline group median for predicted years to onset.

Detailed medical and psychiatric assessments as well as relevant demographic characteristics and family history were obtained from all participants. The Montreal Cognitive Assessment (MoCA) was conducted in order to screen for cognitive impairment. Participants with a score < 26/30 points were excluded to ensure that all participants understand the tasks (Nasreddine et al., 2005). The presence of psychiatric comorbidities like depressive symptoms, suicidal ideations, hallucinations, psychosis, alcohol or illicit drug abuse, apathy, irritability, or aggressive behavior was assessed using semistructural interviews based on the Columbia Suicide Severity Rating Scale (CSSRS) (Posner et al., 2011), the Apathy Evaluation Scale (Marin, Biedrzycki, & Firinciogullari, 1991), and the Problem Behaviors Assessment (PBA) (Craufurd, Thompson, & Snowden, 2001). Participants with major depression, psychosis, alcohol or illicit drug abuse, or physically aggressive behavior were excluded. None of the mutation carriers had any concomitant neuropsychiatric symptoms.

### 2.2 Neuropsychological test battery

#### 2.2.1 Beads task

This is an information sampling task assessing how much information participants gather before making a decision (Djamshidian et al., 2012). For further details, see Djamshidian et al. (2012).

Briefly, the beads task is an information sampling task where two cups are used. One cup contains more green than blue beads and vice versa for the other cup. Participants are shown one bead (either green or blue). They then can decide if they want to draw another bead before making a decision, or they can immediately (or after each bead, respectively) decide from which cup this bead was drawn.

Furthermore, we used two ratios in the beads task. One low conflict 80:20 ratio (blue cup: 80% blue, 20% green beads; green cup: 80% green, 20% blue beads) and one high conflict 60:40 ratio (blue cup: 60% blue, 40% green beads; green cup: 60% green, 40% blue beads). Each ratio was presented six times.

The best strategy is to draw several beads prior to making a decision. Therefore, we were interested in the total number of draws participants made before choosing a cup (“drawing behavior”).

#### 2.2.2 Trail making test A and B (TMT)

Participants were asked to complete the TMT part A and B (Ehrenstein, Heister, & Cohen, 1982) Participants have to draw lines to conflate numbers in ascending order (part A). In part B, participants have to connect lines alternating between numbers and letters in ascending order (i.e., 1‐A‐2‐B‐3‐C; part B). At the end, total time to completion per part was calculated (seconds).

#### 2.2.3 Symbol digit modalities test (SDMT)

The SDMT consists of a page headed by a key that pairs the single digits 1–9 with nine matching symbols (Smith, 1982). The rows below contain only symbols, and participants have to report the correct number for each symbol in the spaces below. After completing the first 10 items with guidance, participants are timed to determine how many responses can be made in 90 s.

### 2.3 Statistics

Statistical analyses were performed using SPSS24.0. Parametric and nonparametric tests were used for statistical analysis depending on the distribution and the scale type of variables.

#### 2.3.1 Beads task

Drawing behavior was calculated using a sum score per ratio (either 60:40 or 80:20). To calculate irrational decision making, the investigators (BH, MP) analyzed total beads colors shown and the chosen cup by the participant, for example, rational decision: two blue beads were drawn, blue cup chosen; for example, irrational decision: two green beads were shown, blue cup chosen. A generalized linear model (Poisson) with a loglinear link function was used. As a dependent variable, we used the number of draws before making a decision and the number of irrational choices. Group (HC, HD, pre‐HD: FAR and NEAR) was modeled as a fixed factor. Gender, age, education, and MoCA scores were used as covariates.

All pairwise comparisons were Bonferroni corrected. A *p*‐value equal or below .05 was considered significant.

#### 2.3.2 Trail making test part A (TMT A) and B (TMT B)

Sum scores of time to completion for TMT A and TMT B were calculated per participant with a maximum of 240 s per task. A univariate general model ANOVA was used. Again group (HC, HD, pre‐HD: FAR and NEAR) was modeled as a fixed factor and gender, MoCA scores, and age were defined as covariates. Post hoc analyses of group differences were realized. All pairwise comparisons were Bonferroni corrected. A *p*‐value equal or below .05 was considered significant.

#### 2.3.3 Symbol digit modalities test (SDMT)

Sum scores of correct answers within 90 s in the SDMT were calculated per participant. A univariate general model ANOVA was used. Group (HC, HD, pre‐HD: FAR and NEAR) was modeled as a fixed factor and gender, MoCA scores, and age were defined as covariates. Post hoc analyses of group differences were realized. All pairwise comparisons were Bonferroni corrected. A *p*‐value equal or below .05 was considered significant.

## 3 Results

Demographic data are summarized in Table 1.

**Table 1:** Demographic data

	HD	NEAR	FAR	HC	*p*‐value
Number	22	16	13	26	‐
Gender (male:female)	9:13	10:6	4:9	17:9	.23
MoCA (±*SD*)	27.2 ± 1	28.3 ± 1.2	28.7 ± 1.3	29.3 ± 1.1	<.001***
Education (years) (±*SD*)	12.1 ± 3.7	15.5 ± 3.0	13.2 ± 3.2	14.9 ± 2.2	.002**
Age (years) (±*SD*)	50.7 ± 9.9	39.7 ± 8.9	33.2 ± 5.1	40.7 ± 11.0	<.001***
CAG‐repeats (±*SD*)	44.4 ± 3.4	43.9 ± 2.9	41.9 ± 1.7	‐	.058
UHDRS‐TMS (±*SD*)	26.9 ± 14.0	3.3 ± 2.1	1.2 ± 1.4	‐	<.001***
YTO Langbehn (years) (±*SD*)	‐	10.9 ± 3.5	22.8 ± 5.6	‐	<.001***
Disease duration (years) (±*SD*)	3.8 ± 2.9	‐	‐	‐	‐
TFC (±*SD*)	11.6 ± 1.2	‐	‐	‐	‐

Abbreviations: CAG, cytosine, adenine, and guanine; FAR, carriers far to disease onset; HC, healthy controls; HD, patients with manifest Huntington's disease; MoCA, Montreal Cognitive Assessment battery; NEAR, carriers near to disease onset; *SD*, standard deviation; TFC, total functional capacity; UHDRS‐TMS, Unified Huntington's Disease Rating Scale Total Motor Score; YTO Langbehn, years to disease onset calculated with the Langbehn formula.

All values are mean ± standard deviation.

* *p*‐value < .05; **p < .01; ***p < .001.

Demographic characteristics of all study participants are shown in Table 1. Years to estimated disease onset were calculated using the Langbehn formula.

### 3.1 Beads task

**Figure 1:**
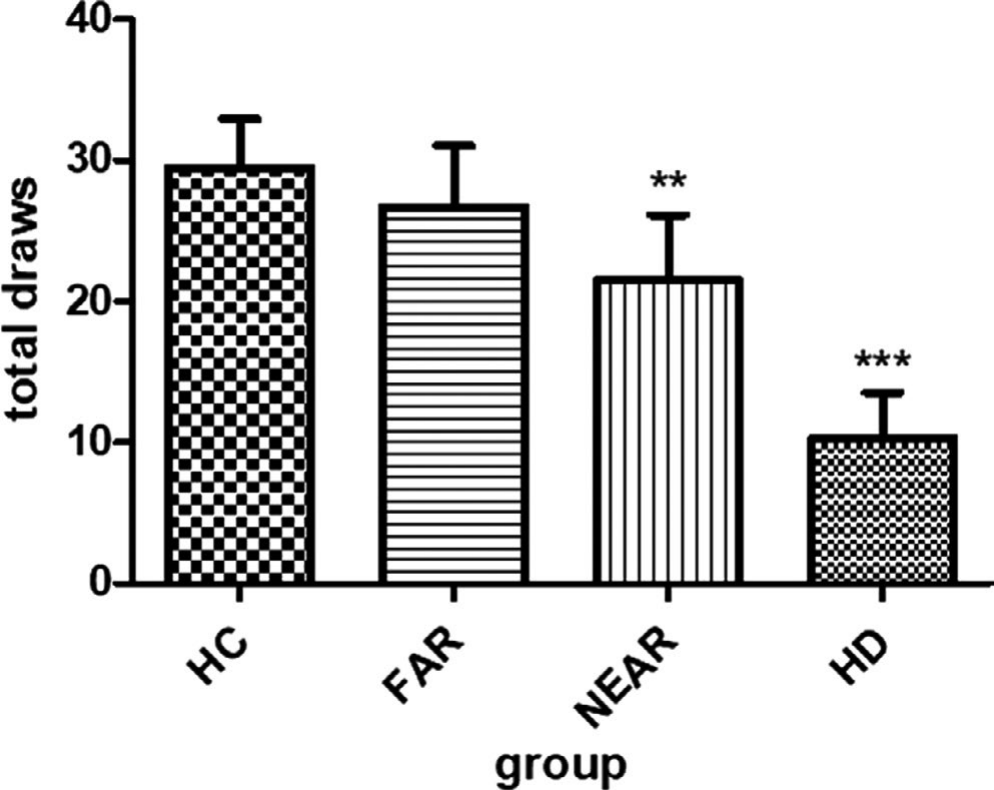
Mean draws in the beads task compared by group. ^*^ *p* < .05; ^***^ *p* < .001; HC, healthy controls; HD, patients with manifest Huntington’s disease; near_pre‐HD, premanifest HD mutation carriers near to estimated disease onset (<15 years); far_pre‐HD, premanifest HD mutation carriers far to estimated disease onset (>15 years)

**Figure 2:**
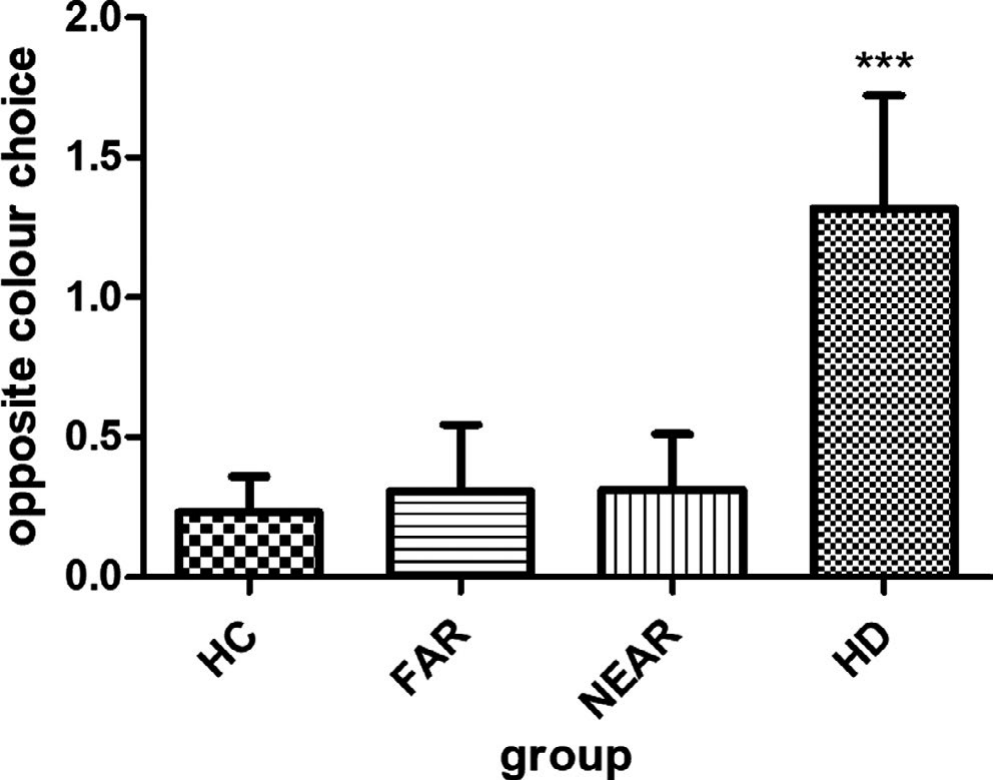
Opposite colour choice in the beads task by group. ^***^ *p* < .001; HC, healthy controls; HD, patients with manifest Huntington’s disease; near_pre‐HD, premanifest HD mutation carriers near to estimated disease onset (<15 years); far_pre‐HD, premanifest HD mutation carriers far to estimated disease onset (>15 years)

In the beads task, HC drew 29.5 (±17.5) times before they made a decision, whereas they only decided 0.4 (±0.8) times against the evidence (Table 2, Figure 1 and Figure 2).

**Table 2:** Neuropsychological tests, Bonferroni corrected

	HD *n* = 22	NEAR *n* = 16	FAR *n* = 13	HC *n* = 26	*p*‐value
Beads task^j^					
Total draws (*n*) ±*SD*	10.3 ± 15.4	21.5 ± 18.6	6.7 ± 15.8	9.5 ± 17.5	<.001***^a^
Irrational decisions (*n*) ±*SD*	13 ± 1.9	0.3 ± 0.8	0.3 ± 0.9	0.4 ± 0.8	.54^b^
TMT A (sec) ±SD^k^	117.9 ± 89.4	22.4 ± 5.5	27.9 ± 14.9	28.8 ± 10.0	<.001***^g^
TMT B (sec) ±SD^k^	209.6 ± 80.1	56.1 ± 29.3	47.9 ± 18.7	53.4 ± 13.2	<.001***^h^
SDMT ± SD^k^	20.7 ± 10.4	50.8 ± 8.2	55.3 ± 7.1	46.9 ± 10.1	<.0001***^i^

Abbreviations: HC, healthy controls; HD, patients with manifest Huntington's disease; NEAR, HD mutation carriers near to calculated disease onset; FAR, HD mutation carriers far to calculated disease onset; *n*, number; RT, reaction time; *SD*, standard deviation; sec, seconds; TMT A, trail making test part A; TMT B, trail making test part B.

All values are mean ± *SD*, except for “errors.”

Post hoc group comparisons were done when a significant main effect for group (*p* < .05) was revealed; all *p*‐values are corrected for age, education, MoCA, gender, and multiple comparisons (Bonferroni):

^a^ HD versus HC, *p* < .001***; HD versus FAR, *p* < .001***; HD versus NEAR, *p* < .001***; HC versus FAR, *p* = 1.0; HC versus NEAR, *p* = .001*** FAR versus NEAR, *p* = .013*.

^b^ HD versus HC, *p* = .92; HD versus FAR, *p* = 1.0; HD versus NEAR, *p* = 1.0; HC versus FAR, *p* = 1.0; HC versus NEAR, *p* = 1.0; FAR versus NEAR, *p* = 1.0.

^g^ HD versus HC, *p* = .007**; HD versus FAR, *p* = .007**; HD versus NEAR, *p* < .001***; HC versus FAR, *p* = 1.0; HC versus NEAR, *p* = 1.0; FAR versus NEAR, *p* = 1.0.

^h^ HD versus HC, *p* < .001***; HD versus FAR, *p* < .001***; HD versus NEAR, *p* < .001***; HC versus FAR, *p* = 1.0; HC versus NEAR, *p* = 1.0; FAR versus NEAR, *p* = 1.0.

^j^ Results of generalized linear model (Poisson) are reported as means ± *SD*.

^k^ Results of mixed model ANOVA are reported as means ± *SD*.

^I^ HD versus HC, *p* < .001***; HD versus FAR, *p* < .001***; HD versus NEAR, *p* < .001***; HC versus FAR, *p* = .143; HC versus NEAR, *p* = .63; FAR versus NEAR, *p* = 1.0.

* *p* < .05; ^**^ *p* < .01; ^***^ *p* < .001.

Looking at the prodromal HD mutation carriers, we found that the NEAR group drew 21.5 (±18.6) beads before choosing a cup, and they made 0.3 (±0.8) irrational decisions. The FAR group drew 26.7 (±15.8) beads and made 0.3 (±0.9) times decisions against the evidence.

The manifest HD group drew least beads of all (10.3, ±15.4) and made 13 (±1.9) irrational decisions.

Altogether, we found that HD patients made decisions with a higher degree of uncertainty than HC and premanifest mutation carriers (all *p*‐values < .001).

Moreover, we found that the NEAR group gathered significantly less information than the FAR group (*p* < .001).

Irrational decision were generally rare in this study, and we did not find any group differences when corrected for age, education, gender, and MoCA (*p* = .54).

There was a significant effect of group (HD patients, FAR, NEAR, HC) (Wald χ^2^ = 42.4, *p* < .001) and beads ratio (60:40; 80:20) (Wald χ^2^ = 122, *p* < .001). In other words, all participants drew more beads in the high conflict (60:40) than in the low conflict (80:20) condition.

HD patients gathered less information than all other groups in the 60:40 (*p* < .001) and less than the HC and the FAR group in the 80:20 ratio (*p* < .001).

Furthermore, the NEAR group sampled less information in the 60:40 ratio (*p* = .029) and in the 80:20 ratio less beads compared to HC (*p* = .037) and the FAR group (*p* < .001).

### 3.2 Trail making test

The test was performed in the majority of participants (18/22 (82%) HD patients, 23/26 (88%) HC), and all premanifest HD mutation carriers (Table 2). HD patients performed significantly worse than all other groups (all *p*‐values < .01), but there were no other group differences (all *p*‐values = 1.0).

### 3.3 Symbol digit modalities test

The test was performed in the majority of participants 18/22 (82%) HD patients, 23/26 (88%) HC), and all premanifest HD mutation carriers (Table 2). HD patients performed significantly worse than all other groups (all *p*‐values < .01), but there were no other significant group differences (all *p*‐values > .1).

Table 2 shows the mean results of neuropsychological tests per group.

Figure 1 shows mean beads drawn in the beads task per group prior to making a decision (e.g. blue or green cup).

Figure 2 shows mean of opposite choices in the beads task made per group (e.g., blue bead shown, green cup chosen).

### 3.4 Discussion

In this study, we investigated cognitive performance in HD patients and HD mutation carriers “FAR” (>15 years) and “NEAR” (<15 years) to estimated disease onset using the beads task, which is an information sampling task. The aim of this study was to assess whether premanifest HD mutation carriers show deficits in decision making and whether there is a difference between HD mutation carriers near and far to estimated disease onset.

We found that manifest HD patients made decisions with a higher degree of uncertainty than all other groups. Moreover, NEAR mutation carriers gathered significant less information than the FAR group and HC, whereas there was no difference between the FAR group and HC.

Irrational decision was generally rare in this study. It is, however likely, that a larger sample size would have revealed group differences.

Both HD patients and NEAR carriers drew fewer beads in the low and in the high conflict trials than the FAR carriers or HC. This behavior with a lack of risk stratification is a sign of reflection impulsivity (Kelland & Lewis, 1994), encompassing impulsivity and poor risk assessment (Della Nave et al., 2010; Hamilton, 2003). Jumping to conclusions is typically found in patients with psychotic disorders and substance abusers and patients with behavioral addictions (Djamshidian, O'Sullivan, et al., 2013; Djamshidian, Sanotsky, et al., 2013; Dudley et al., 2016; So et al., 2016).

In order to avoid potentially confounding factors that could lead to impairment in decision making, we excluded participants with apathy. However, there are also data that patients with neurodegenerative diseases perform better in specific tasks than expected due to increased motivation toward receiving rewards, like it is in the beads task (Czernecki, Houeto, Pochon, Levy, & Dubois, 2002; Perry et al., 2015).

The PREDICT‐HD study addresses that prodromal HD patients close to receiving a motor diagnosis show numerous differences from controls in cognitive tasks including TMT, SDMT, Stroop, and Letter Fluency (Stout et al., 2011). In this study, they stratified prodromal HD mutation carriers into a NEAR (≤9 years to estimated disease onset), a MID (between 9 and 15 years to estimated disease onset), and a FAR (≥15 years to estimated disease onset) group. They used nineteen cognitive tasks to assess working memory, attention, language, psychomotor functions, episodic memory, recognition of facial emotion, and executive functions. They could show that the NEAR group performed significantly poorer than HC and that symptoms are more difficult to detect in those carriers far from diagnosis.

Another study assessing psychomotor and executive functioning in preclinical HD could show a difference between carriers close to clinical onset of HD and those many years from HD onset (Snowden et al., 2002). Functional magnetic imaging studies have shown that the beads task activates a wide network including the ventral striatum, the anterior cingulate, the parietal cortex, and the insula (Della Nave et al., 2010; Enzi et al., 2012; Furl & Averbeck, 2011; Hamilton, 2003; Seo et al., 2012). These brain areas are also typically affected in patients with HD and also in asymptomatic mutation carriers. Previous studies in premanifest mutation carriers have demonstrated striatal hypometabolism (Lopez‐Mora et al., 2016), white matter changes in the striatum, the corpus callosum, and in posterior white matter tracts (Della Nave et al., 2010; Muhlau et al., 2007), as well as cortical thinning which significantly correlates with motor and cognitive decline (Sweidan et al., 2020). A previous PET study could show a decreased expression PDE10A, an important biomarker for HD, which is highly expressed in medium spiny neurons, in the striatum and pallidum and an increased expression in the motor thalamus of premanifest HD gene carriers compared to healthy controls (Niccolini et al., 2015). Functional PET studies using (11C) IMA107 demonstrated a 25%–33% reduction in striatal PDE10A 25 years prior to predicted symptomatic onset (Niccolini et al., 2015; Wilson et al., 2016).

Interestingly, there are studies suggesting that due to the association of the SDMT and the TMT, speed of perceptual processing and visual search are major components of the TMT, and prodromal changes of the occipital cortex may explain this early salience in motoric asymptomatic gene carriers (Lange, 1981; Rosas et al., 2008). In line with that, an imaging study demonstrated an inverse correlation between cortical atrophy in the occipital lobe and performances in the SDMT and Stroop task (Rosas et al., 2008). It is therefore assumed that prodromal occipital degeneration occurs prior to motor symptoms onset in HD, similar to what has been observed in the basal ganglia (Aylward et al., 2004).

Nevertheless, we found that HD patients were slower than all other groups in the TMT and SDMT, but in contrast to previous studies, there was neither a difference between the NEAR and the FAR group, nor between the pre‐HD cohort and HC (Hart et al., 2011; Keuken et al., 2014; Kirkwood, 2000; Matsui et al., 2014; McGarry & Biglan, 2017). There are several explanations for this discrepancy: First, in our study only a minority of our NEAR pre‐HD patients (8/16) was “very near” to estimated disease onset (≤9 years); second, the sample size in the pre‐HD group was relatively small and it is possible that a larger sample size would have demonstrated significant group differences.

Finally, previous studies showed that the beads task was highly sensitive to detect impairment in decision making in various patient groups (Averbeck et al., 2013; Averbeck, O'Sullivan, & Djamshidian, 2014; Djamshidian et al., 2012; Heim et al., 2016). Therefore, we speculated that the beads task is more sensitive to detect early changes in frontostriatal circuits than the TMT or the SDMT and therefore detects earlier and more subtle changes in these brain areas.

Summarizing, we found that manifest HD patients gathered significantly less evidence than all other groups and showed poorer cognitive flexibility. Mutation carriers far from estimated disease onset performed equally to HC and better on the beads task than mutation carriers near to estimated disease onset. Furthermore, we conclude that jumping to conclusions is an early marker of cognitive dysfunction in premanifest HD patients prior to reaching criteria for the motor diagnosis of HD.

This neuropsychological sign can possibly improve diagnostic prediction of the disease.

## CONFLICT OF INTEREST

B.H. reports no disclosures related to the submitted work. B.H. reports personal fees from Novartis and travel grants from the International Parkinson's disease and Movement Disorder Society and the Austrian Parkinson´s Disease Society outside the submitted work. M.P. reports no disclosures related to the submitted work. M.P. reports travel grants from the International Parkinson's disease and Movement Disorder Society and the Austrian Parkinson´s Disease Society outside the submitted work. C.S. reports no disclosures related to the submitted work. C.S. reports personal fees from honorarium from TEVA GmbH, grants from Biogen and the “Cure Huntington's Disease Initiative” (CHDI), other from Institutional compensation and/or travel or accommodation payments in the context of the ENROLL‐HD study (CHDI), the ACR16‐Study (NeuroSearch), the AFQ‐Study (Novartis), the Selisistat‐Studies (Siena Biotech), the PRIDE‐ and LEGATO‐HD‐Studies (TEVA), the Amaryllis‐Study (Pfizer), Ipsen, Antisense‐Study (IONIS/ Roche AG), all outside the submitted work. S.M.H. reports no disclosures related to the submitted work. S.M.H. reports grants from the “Cure Huntington's Disease Initiative” (CHDI), other from Institutional compensation and/or travel or accommodation payments in the context of the ENROLL‐HD study (CHDI), all outside the submitted work. P.E. reports no disclosures related to the submitted work. B.H. reports travel grants from the International Parkinson's disease and Movement Disorder Society and the Austrian Parkinson´s Disease Society outside the submitted work. J.M.P. has nothing to declare. K.S. reports no disclosures related to the submitted work. K.S. reports personal fees from Biogen, Teva, UCB, Lundbeck, AOP Orphan Pharmaceuticals AG, Roche, Grünenthal and Abbvie, honoraria from the International Parkinson and Movement Disorders Society, research grants from FWF Austrian Science Fund, Michael J. Fox Foundation, and International Parkinson and Movement Disorder Society, outside the submitted work. A.D. reports no disclosures related to the submitted work.

## AUTHOR CONTRIBUTIONS

BH contributed to the conception of the study, analysis and interpretation of the data, and drafting the manuscript. MP contributed to data collection and review of the manuscript. CS contributed to analysis and interpretation of the data, contributed to data collection, and review of the manuscript. SMVH contributed to analysis and interpretation of the data, contributed to data collection, and review of the manuscript. JMP contributed to data collection and review of the manuscript. PE contributed to drafting and review of the manuscript. KS contributed to conception of the study, interpretation of the data, and review of the manuscript. AD contributed to the conception of the study, analysis and interpretation of the data, and drafting the manuscript.

### Peer Review

The peer review history for this article is available at https://publons.com/publon/10.1002/brb3.1843.

## Data Availability

The data that support the findings of this study are available from the corresponding author upon request.
